# Surgical treatment of pulmonary hydatidosis: six years of experience and results at the National Hospital Guillermo Almenara Irigoyen, Lima, Peru

**DOI:** 10.17843/rpmesp.2026.433.15699

**Published:** 2026-09-18

**Authors:** Ricardo Taipe-Mallqui, Georgette Vetanzo-Sánchez, Daniel Karl Ernesto Muratta Pareja, José Manuel Palacios León, Fernando Valdivia Mamani, Jorge Cervera Farfan

**Affiliations:** 1 Departamento de Cirugía de Tórax, Hospital Nacional Guillermo Almenara Irigoyen, Lima, Peru. Departamento de Cirugía de Tórax Hospital Nacional Guillermo Almenara Irigoyen Lima Peru; 2 Universidad Nacional Mayor de San Marcos, Lima, Peru. Universidad Nacional Mayor de San Marcos Universidad Nacional Mayor de San Marcos Lima Peru; 3 Universidad Privada Antenor Orrego, La Libertad, Peru. Universidad Privada Antenor Orrego Universidad Privada Antenor Orrego La Libertad Peru

**Keywords:** Pulmonary Echinococcosis, Pulmonary Surgical Procedures, Peru

## Abstract

The objective was to describe the clinical and perioperative characteristics of patients undergoing surgery for pulmonary hydatidosis at the Guillermo Almenara Irigoyen National Hospital (HNGAI). An observational, descriptive, and retrospective study was conducted that included 62 patients with pulmonary hydatid cyst (PHC) who underwent surgery between 2019 and 2024. The mean age was 39.0 ± 17.8 years, and 51.6% of the patients came from inland regions of the country. The mean diameter of the cysts was 9.3 ± 3.4 cm; 66.1% presented with perihilar location, and 40.3% with multiple cysts. Lobectomy was the most frequent surgical procedure (59.7%). Postoperative complications occurred in 12.9% of patients, and no perioperative mortality was recorded. In conclusion, pulmonary hydatidosis was characterized by a significant proportion of perihilar, multiple, and large cysts. Lobectomy was the most used surgical procedure, with a low frequency of postoperative complications.

## INTRODUCTION

Hydatidosis or cystic echinococcosis is a parasitic zoonosis caused by the larval form *of Echinococcus granulosus* and constitutes a public health problem, especially in livestock regions ^1^. Humans act as accidental intermediate hosts after ingesting parasite eggs, whose oncospheres cross the intestinal mucosa and disseminate via hematogenous or lymphatic routes. Cysts are mainly located in the liver (70-85%) and, secondly, in the lungs (15-25%) ^1^^,^^2^. Pulmonary hydatid cyst (PHC) can reach a large size and, although some patients remain asymptomatic, its growth can produce cough, chest pain, dyspnea, and hemoptysis ^1^^-^^4^.

Currently, the persistence of this disease reflects limitations in sanitation control programs and maintains an endemic transmission cycle that affects more than one million people, mainly in Eurasia, the Middle East, and South America ^4^^,^^5^. According to the Pan American Health Organization (PAHO), during 2022-2023, 11213 human cases were confirmed in the region, of which Peru accounted for 76% (8535 cases) ^5^. These results place Peruvian territory as the main hyperendemic focus of the continent, where sociocultural factors, informal livestock farming, and limited access to basic rural health services favor the persistence of the disease.

The treatment of PHC continues to be a challenge. Benzimidazoles, such as albendazole, show limited efficacy and cure rates below 30% ^2^^,^^6^. Therefore, surgery constitutes the treatment of choice for large, symptomatic, or complicated cysts ^1^^-^^6^. Unlike hepatic localization, surgery for PHC does not have a standardized surgical strategy ^2^^,^^5^^,^^6^, due to the mechanical properties of the lung parenchyma, which allow for rapid and silent growth, and the high susceptibility of the cyst to complicate by rupture ^5^^,^^6^. This generates a marked technical heterogeneity, from conservative procedures such as cystectomy plus capitonnage to major pulmonary resections in cases of tissue destruction, hilar localization, or rupture ^(^^6^^-^^11^.

PHC surgery may be associated with postoperative complications (POC), including air leak, bronchopleural fistula, empyema, pneumothorax, and anaphylactic reactions. In Peru, POC were reported in 28.2% and 22.1% of patients in studies published in 2006 and 2008, respectively ^11^, with the most frequent being atelectasis, postoperative hemorrhage, and prolonged air leak. Internationally, giant cysts, rupture, and pulmonary resections have been associated with a higher risk of POC ^12^^,^^13^, while surgical site infection, empyema, and prolonged air leak have been related to longer hospital stays ^14^. In this regard, the available national evidence is limited and comes from studies published two decades ago, which generates a knowledge gap regarding the clinical characteristics, surgical management, and perioperative outcomes of patients currently treated in the country.

Therefore, the objective of this study was to describe the clinical and perioperative characteristics of patients undergoing surgery for pulmonary hydatidosis at the Guillermo Almenara Irigoyen National Hospital (HNGAI), Lima, Peru, during a six-year period.

**KEY MESSAGES**

Motivation for the study. Pulmonary hydatidosis continues to be a relevant problem in Peru; however, there is limited recent national evidence on the characteristics of patients undergoing surgery and their outcomes.

Main findings. In 62 patients operated on between 2019 and 2024, perihilar, multiple, and large cysts were frequent. Lobectomy was the most commonly used surgical procedure. Postoperative complications occurred in 12.9%, and no perioperative deaths were recorded.

Public health implications. These findings show the importance of strengthening timely detection and referral of patients with pulmonary hydatidosis, to facilitate their specialized evaluation and treatment.

## THE STUDY

An observational, descriptive, and retrospective study was conducted, including 62 patients who underwent surgery for pulmonary hydatid cyst at the Thoracic Surgery Service of HNGAI, between January 2019 and December 2024. All patients who met the eligibility criteria during the study period were included.

Patients 14 years or older with a diagnosis of pulmonary hydatidosis and an indication for surgical treatment were included. Pregnant patients, those with hepatic cysts with involvement of the thoracic wall or the mediastinum, and those whose medical records were incomplete or did not contain information on the main study variables were excluded.

The information was obtained by reviewing medical records using a data collection form. Sociodemographic and clinical-radiological variables were recorded, such as age, sex, medical history, place of origin, main symptom, cyst size and rupture, affected lung and lobe, number of cysts, and perihilar location. Perioperative variables included surgical technique, according to lung parenchyma preservation or resection, reoperation, postoperative use of albendazole, postoperative complications, and hospital stay.

Five variables of interest were operationally defined: 1) cyst size, defined as the maximum cyst diameter, expressed in centimeters, measured in the axial plane of the mediastinal window of computed tomography (CT); 2) cyst rupture, defined as the presence of hydroaeric level or floating membranes on chest CT, with intraoperative confirmation; 3) perihilar cyst, defined as a lesion with involvement of vascular or bronchial structures of the pulmonary hilum, identified by CT and corroborated as an intraoperative finding; 4) postoperative complications, defined as any adverse event related to pulmonary surgery recorded during the postoperative period, such as bronchopleural fistula, postoperative bleeding, or empyema; and 5) total hospital stay, defined as the time elapsed from hospital admission until medical discharge, regardless of the admission pathway (outpatient, emergency, or referral) or prior stay in other hospital services, such as internal medicine or pulmonology.

Categorical variables were summarized using absolute frequencies and percentages. For continuous variables, distribution was assessed using the Shapiro-Wilk test and summarized using mean and its standard deviation (SD) or median and interquartile range (IQR). Statistical analysis was performed with Stata BE, version 18.0 (StataCorp, College Station, TX, USA).

The study was approved by the Research Ethics Committee of HNGAI (Letter No. 118 CIEI-OIyD-GRPA-ESSALUD-2025). The development and submission of the manuscript followed the recommendations of the guide Strengthening the Reporting of Observational Studies in Epidemiology (STROBE) ^15^.

## FINDINGS

A total of 69 patients with a diagnosis of pulmonary hydatidosis underwent surgery in the Thoracic Surgery Department of HNGAI. Seven patients were excluded due to incomplete medical records or for not meeting the eligibility criteria, so the analysis finally included 62 patients. The mean age was 39.0 ± 17.8 years and 58.1% (36/62) had no relevant pathological history (table 1). Sex distribution was similar, with 31 men (50.0%) and 31 women (50.0%). Regarding the place of origin, 51.6% (32/62) came from regions in the interior of the country, while 48.4% (30/62) came from Lima.

**Table 1:** Preoperative characteristics of patients diagnosed with pulmonary hydatidosis who underwent pulmonary surgery at the Hospital Nacional Guillermo Almenara Irigoyen during the period 2019-2024.

Preoperative characteristics		n (%)
Age, mean (±SD)		39.0 ± 17.8
	14-24 years	15 (24.2)
	25-49	29 (46.8)
	50-64	12 (19.3)
	65 or more	6 (9.7)
Sex		
	Female	31 (50.0)
	Male	31 (50.0)
Background		
	None	36 (58.1)
	Diabetes mellitus	1 (1.6)
	Hypertension	3 (4.8)
	Surgery for pulmonary hydatid cyst	6 (9.7)
	Hepatic hydatid cyst	7 (11.3)
	Others	9 (14.5)
Affiliation		
	Lima	30 (48.4)
	Provinces	32 (51.6)
Main Symptom		
	Asymptomatic	3 (4.8)
	Chest pain	10 (16.1)
	Dyspnea	13 (21.0)
	Vomica cough	23 (37.1)
	Hemoptysis	11 (17.7)
	Fever	2 (3.2)
Maximum diameter (cm) of the cyst, mean (±SD)		9.3 ± 3.4
	≤10	37 (59.7)
	>10	25 (40.3)
Rupture		
	No	45 (72.6)
	Yes	17 (27.4)
Affected Lung		
	Right	40 (64.5)
	Left	22 (35.5)
Number of cysts		
	Unique	37 (59.7)
	Multiple	25 (40,3)

SD: standard deviation, cm: centimeters.

The most frequent preoperative symptom was cough with vomica, present in 37.1% (23/62) of the patients. Regarding the characteristics of the cysts, the right lung was the most affected 64.5%; (40/62). 59.7% (37/62) of the patients presented a single cyst and 40.3% (25/62), multiple cysts. Likewise, 59.7% (37/62) presented cysts with a diameter ≤10 cm, while 40.3% (25/62) presented cysts >10 cm. At the time of diagnosis, 27.4% (17/62) presented cyst rupture (table 1).

Lobectomy was the most frequent surgical procedure, performed in 59.7% (37/62) of patients. Among the surgical findings, the right lower lobe was the most frequent location of PHC, present in 33.9% (21/62) of cases. Likewise, perihilar location of the cyst was documented in 66.1% (41/62) of patients (figures 1 and 2).

**Figure 1:**
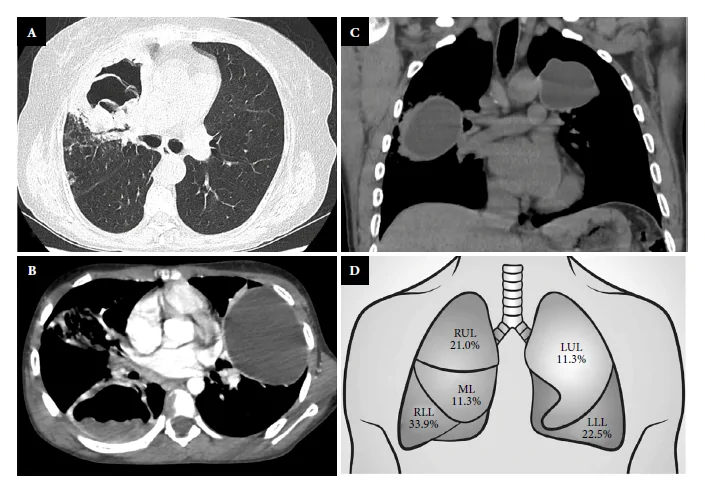
Tomographic findings and anatomical distribution of pulmonary hydatid cyst. A) Axial tomography (pulmonary window) showing a ruptured cyst with collapsed membranes (water lily sign). B) Axial tomography (mediastinal window) with multiple presentation. C) Coronal reconstruction highlighting the bilateral perihilar location and its relationship with the mediastinum. D) Scheme of the percentage distribution according to the affected lobe (RUL: right upper lobe; ML: middle lobe; RLL: right lower lobe; LUL: left upper lobe; LLL: left lower lobe).

**Figure 2:**
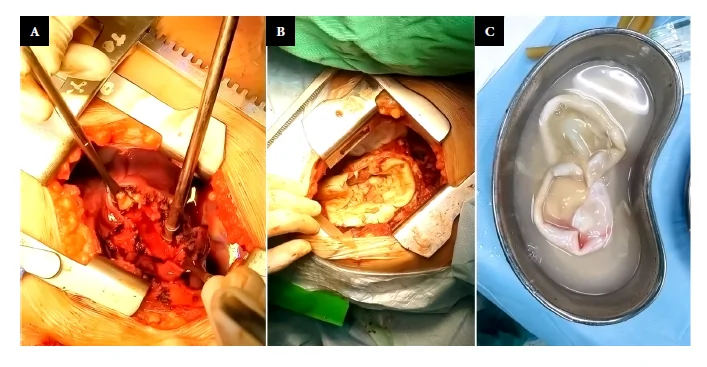
Intraoperative findings of surgical specimens for pulmonary hydatid cyst. A) Intraoperative approach by thoracotomy showing the opening of the pulmonary cavity and the aspiration of the hydatid cyst content. B) Exposure of the collapsed residual cavity after opening the perikystic layer. C) Whitish germinative and cuticular hydatid membrane.

During the postoperative period, 75.8% (47/62) of patients received complementary treatment with albendazole. Postoperative complications occurred in 12.9% (8/62) of patients, with bronchopleural fistula and surgical site infection being the most frequent. 14.5% (9/62) required reoperation mainly due to hemorrhage, bronchopleural fistula, or empyema. One patient with bronchopleural fistula required two reoperations: one for fistula closure and another for empyema management. The median total hospital stay was 12 days (IQR: 6-23 days). No perioperative deaths related to pulmonary hydatid cyst surgery were recorded (table 2).

**Table 2:** Postoperative characteristics of patients diagnosed with pulmonary hydatidosis who underwent pulmonary surgery at Hospital Nacional Guillermo Almenara Irigoyen during the 2019-2024 period.

Postoperative Characteristics		n (%)
Surgical technique		
	Lobectomy	37 (59.7)
	Cystectomy + capitonnage	22 (35.5)
	Lobectomy + cystectomy	3 (4.8)
Affected pulmonary lobe		
	Right superior lobe	13 (21.0)
	Middle lobe	7 (11.3)
	Right inferior lobe	21 (33.9)
	Left superior lobe	7 (11.3)
	Left inferior lobe	14 (22.5)
Perihilar cyst		
	No	21 (33.9)
	Yes	41 (66.1)
Reoperation		
	No	53 (85.5)
	Yes	9 (14.5)
Use of postoperative albendazole		
	No	15 (24.2)
	Yes	47 (75.8)
Complication		
	No	54 (87.1)
	Yes	8 (12.9)
Type of complication		
	None	54 (87.1)
	Bronchopleural fistula	2 (3.2)
	Empyema	1 (1.6)
	Surgical site infection	2 (3.2)
	Bleeding	1 (1.6)
	Sepsis	2 (3.3)
Hospitalization days, median and interquartile range		12 (6-23)

## DISCUSSION

In this study, pulmonary hydatidosis mainly affected young adults, with a mean age of 39.0 ± 17.8 years and a predominance of patients without relevant medical history. These findings are comparable to those reported at the Cayetano Heredia National Hospital, where the mean age was 32.9 ± 18.6 years ^(^^16^, and at the Regional Clinical Hospital of Concepción, Chile, where it was 34.4 ± 19.0 years ^9^, although they differ from what was described at the Dos de Mayo National Hospital, with a mean of 21.6 ± 14.1 years ^10^^,^^11^.

No differences were observed in sex distribution, with an equal proportion of men and women. This result is similar to other national studies, in which the proportion of men ranged between 50.4% and 54.0% ^10^^,^^11^, but differs from some international series, where a male predominance of up to 62.7% ^(12-14)^ has been reported. Regarding the place of origin, 51.6% of patients came from regions in the interior of the country and 48.4% from Lima, whereas previous national studies reported that between 77.0% and 79.0% of cases came from rural areas and had a history of indoor dog breeding (10,11,16). This suggests that the patients currently residing in Lima correspond to cases of internal migration from endemic areas.

It is important to note that a proportion of patients residing in Metropolitan Lima had contact with high Andean and endemic regions of Peru, such as Huánuco, Pasco, Junín, Huancavelica, Apurímac, Cusco, Arequipa, and Puno. This pattern is compatible with the influence of population mobility from endemic areas to Lima; however, since detailed information on previous residence, duration of exposure, contact with dogs, or livestock activities was not available, it is not possible to establish the probable place or time of infection acquisition. These findings highlight the importance of strengthening hydatidosis prevention and control measures in endemic areas ^10^^,^^11^^,^^16^.

Regarding the clinical presentation, cough and vomica were the predominant symptoms. Additionally, 27.4% of the patients presented with cyst rupture at the time of diagnosis. The rupture of the PHC can cause communication with the bronchial tree and trigger a local inflammatory response, which favors the appearance of manifestations such as cough, vomica, chest pain, and hemoptysis ^17^^,^^18^. The proportion of ruptured cysts observed in our series could be related to larger lesions or with a prolonged evolution before treatment.

The mean cyst diameter was 9.3 ± 3.4 cm, similar to that reported in a Chilean series (9.1 ± 4.9 cm) ^9^ and greater than that described in Turkish series, where it ranged between 4.0 and 6.0 cm ^12^. Although the majority of patients presented a single cyst (59.7%), consistent with previous national studies ^10^^,^^11^, 40.3% had multiple cysts, which may be related to different dissemination mechanisms or parasite exposure and constitutes a clinically relevant aspect due to the greater complexity it may represent for the therapeutic approach ^(^^2^^,^^10^^,^^11^^,^^19^.

From a surgical perspective, lobectomy was the most frequent procedure (59.7%), in agreement with previous national series ^10^^,^^11^, but contrasts with reports from the Middle East and South America, where conservative approaches such as cystectomy with capitonnage predominate (83.7%) ^8^^,^^9^^,^^20^. This need to resort to major resections in our setting reflects a higher proportion of complex, perihilar, ruptured cysts or those with parenchymal destruction that prevents lung preservation ^10^^,^^11^^,^^16^^,^^21^, concentrating mainly in the right lower lobe (33.9%) due to anatomical and pulmonary perfusion factors ^2^^,^^6^^,^^22^.

Far from being a strictly intraoperative technical challenge, this high proportion of complex forms constitutes a direct reflection of a latent public health problem linked to late diagnosis, delayed referrals, and internal migration patterns in the country. Faced with this scenario, the Peruvian health system requires a transition from a purely recuperative approach towards timely detection strategies that begin from school age and adolescence in endemic areas. Likewise, in urban areas, the implementation of epidemiological screening protocols through chest radiography or ultrasound in primary care and in annual occupational health evaluations is important, focused on people with a history of residence or livestock exposure in their youth. This intervention would allow early detection of PHC, reducing the number of resective pulmonary surgeries ^5^^,^^23^, shortening hospital stay, and mitigating the negative socioeconomic impact that this disease generates on the economically active population of social security.

Regarding postoperative management, 75.8% of patients received albendazole as complementary treatment, in accordance with the use of benzimidazoles in certain scenarios of cystic echinococcosis ^7^^,^^23^. The remaining 24.2% did not receive albendazole due to the presence of ruptured PHC with evacuation of the membranes before the surgical procedure, a scenario in which the benefit of antiparasitic treatment would be limited ^2^^,^^5^^,^^7^^,^^23^. On the other hand, postoperative complications occurred in 12.9% of patients, a proportion lower than the 15.3 to 27.1% reported in other series ^10^^,^^11^^,^^24^^,^^25^. These favorable results from our study should be interpreted considering the descriptive nature and the center’s experience in the surgical management of PHC.

This study presents some limitations inherent to its observational, descriptive, and retrospective design, based on the review of medical records. Firstly, the availability and quality of clinical and radiological records could lead to information bias. Furthermore, detailed information was not available on potential exposure factors related to disease transmission, such as time of residence in rural or endemic areas, cohabitation with dogs, and exposure to livestock activities, which limited a better epidemiological characterization of the patients. Secondly, as this was a study conducted at a single referral center, its results would not be generalizable to patients treated at other institutions. Although all eligible patients during the study period (n=62) were included, the sample size limited the possibility of exploring some associations. Finally, the absence of a standardized surgical protocol during the evaluated period could introduce variability in the selection of the surgical technique and in perioperative outcomes. Future multicenter studies with analytical designs could compare the results of different surgical strategies, particularly lobectomy and conservative procedures, and identify factors associated with the choice of surgical technique and the occurrence of postoperative complications.

In conclusion, pulmonary hydatidosis in our series primarily affected young adults, mostly without relevant pathological history, and was characterized by a significant proportion of perihilar, multiple, and large cysts. Lobectomy was the most frequently used surgical procedure, with a low frequency of postoperative complications and absence of perioperative mortality. These findings reaffirm the need to optimize timely referral mechanisms to specialized centers, implement screening programs aimed at the young population in endemic areas, and incorporate periodic occupational evaluations in urban environments receiving migration.

## References

[1] Craig PS, McManus DP, Lightowlers MW, Chabalgoity JA, Garcia HH, Gavidia CM (2007). Prevention and control of cystic echinococcosis. Lancet Infect Dis.

[2] Wen H, Vuitton L, Tuxun T, Li J, Vuitton DA, Zhang W (2019). Echinococcosis: advances in the 21st century. Clin Microbiol Rev.

[3] Deplazes P, Rinaldi L, Alvarez Rojas CA, Torgerson PR, Harandi MF, Romig T (2017). Global distribution of alveolar and cystic echinococcosis. Adv Parasitol.

[4] McManus DP, Gray DJ, Zhang W, Yang Y (2012). Diagnosis, treatment, and management of echinococcosis. BMJ.

[5] Centro Panamericano de Fiebre Aftosa y Salud Pública Veterinaria (2020). Programa Regional para la eliminación de la equinococosis quística/hidatidosis 2020-2029.

[6] Aydin Y, Ulas AB, Dostbil A (2025). Current management of pulmonary hydatid cyst. Eurasian J Med.

[7] World Health Organization (2025). WHO guidelines for the treatment of patients with cystic echinococcosis.

[8] Imran M, Abid Khan M, Janas I, Ullah M, Ullah H, Baseer A (2025). Efficacy and safety of surgical techniques in the management of pulmonary hydatid disease: a retrospective cohort study. Cureus.

[9] González L R, Riquelme U A, Reyes M R, Alarcón O F, Spencer L L, Barra M S (2020). Hidatidosis pulmonar características, manifestaciones clínicas y tratamiento en pacientes hospitalizados en Concepción, Región del Biobío, Chile. Rev Med Chil.

[10] Rafael A, Ramos W, Peralta J, Rojas L, Montesinos E, Ortega-Loayza AG (2008). Hidatidosis pulmonar en un hospital de Lima, Perú experiencia en 113 pacientes. Rev Peru Med Exp Salud Publica.

[11] Vasquez JC, Montesinos E, Peralta J, Rojas L, DeLaRosa J, Leon JJ (2009). Need for lung resection in patients with intact or ruptured hydatid cysts. Thorac Cardiovasc Surg.

[12] Ahmadinejad M, Hashemi M, Azizallahi N (2020). Evaluation of prognostic factors associated with postoperative complications following pulmonary hydatid cyst surgery. Open Respir Med J.

[13] Halezeroglu S, Celik M, Uysal A, Senol C, Keles M, Arman B (1997). Giant hydatid cysts of the lung. J Thorac Cardiovasc Surg.

[14] Ershadi R, Salehi M, Roostaei G, Khoshnam Rad N, Soltanmohammadi S, Amini H (2025). Factors associated with hospital length of stay in patients with thoracic hydatid cyst disease undergoing surgical intervention a retrospective study. J Cardiothorac Surg.

[15] Vandenbroucke JP, von Elm E, Altman DG, Gøtzsche PC, Mulrow CD, Pocock SJ (2014). Strengthening the Reporting of Observational Studies in Epidemiology (STROBE) explanation and elaboration. Int J Surg.

[16] Amez Olivera JM, Castañeda Saldaña E (2002). Tratamiento quirúrgico de la hidatidosis pulmonar en el Hospital Nacional Cayetano Heredia 1989-1999. Rev Med Hered.

[17] Kuzucu A, Soysal O, Ozgel M, Yologlu S (2004). Complicated hydatid cysts of the lung clinical and therapeutic issues. Ann Thorac Surg.

[18] Ozyurtkan MO, Kocyigit S, Cakmak M, Ozsoy IE, Balci AE (2009). Case report secondary pleural hydatidosis. Turkiye Parazitol Derg.

[19] Usluer O, Ceylan KC, Kaya S, Sevinc S, Gursoy S (2010). Surgical management of pulmonary hydatid cysts is size an important prognostic indicator?. Tex Heart Inst J.

[20] Kavukcu S, Kilic D, Tokat AO, Kutlay H, Cangir AK, Enon S (2006). Parenchyma-preserving surgery in the management of pulmonary hydatid cysts. J Invest Surg.

[21] Castillo-Minaya KY, Cherre-Fiestas AC, Somocurcio-Peralta JR (2018). Coexistencia de aspergilosis e hidatidosis pulmonar reporte de un caso. Rev Peru Med Exp Salud Publica.

[22] Xanthakis D, Efthimiadis M, Papadakis G, Primikirios N, Chassapakis G, Roussaki A (1972). Hydatid disease of the chest report of 91 patients surgically treated. Thorax.

[23] Bagheri R, Haghi SZ, Amini M, Fattahi AS, Noorshafiee S (2011). Pulmonary hydatid cyst analysis of 1024 cases. Gen Thorac Cardiovasc Surg.

[24] Saygi A, Oztek I, Guder M, Sungun F, Arman B (1997). Value of fibreoptic bronchoscopy in the diagnosis of complicated pulmonary unilocular cystic hydatidosis. Eur Respir J.

[25] Dakak M, Caylak H, Kavakli K, Gozubuyuk A, Yucel O, Gurkok S (2009). Parenchyma-saving surgical treatment of giant pulmonary hydatid cysts. Thorac Cardiovasc Surg.

