# Supplementary material for: Surgical treatment of pulmonary hydatidosis: six years of experience and results at the National Hospital Guillermo Almenara Irigoyen, Lima, Peru
Source: Rev Peru Med Exp Salud Publica. 2026 Sep 18;43(3):364–70. doi: 10.17843/rpmesp.2026.433.15699 (PMC13718559; doi:10.17843/rpmesp.2026.433.15699)
Supplement: Translation

# Translation: Tratamiento quirúrgico de la hidatidosis pulmonar: seis años de experiencia y resultados en el Hospital Nacional Guillermo Almenara Irigoyen, Lima, Perú

**Authors:** Ricardo Taipe-Mallqui, Georgette Vetanzo-Sánchez, Daniel Karl Ernesto Muratta Pareja, José Manuel Palacios León, Fernando Valdivia Mamani, Jorge Cervera Farfan

**Affiliations:** 1 Departamento de Cirugía de Tórax, Hospital Nacional Guillermo Almenara Irigoyen, Lima, Perú.; 2 Universidad Nacional Mayor de San Marcos, Lima, Perú.; 3 Universidad Privada Antenor Orrego, La Libertad, Perú.

**Keywords:** Equinococosis Pulmonar, Procedimientos Quirúrgicos Pulmonares, Perú

## Abstract

El objetivo fue describir las características clínicas y perioperatorias de los pacientes sometidos a cirugía por hidatidosis pulmonar en el Hospital Nacional Guillermo Almenara Irigoyen (HNGAI). Se realizó un estudio observacional, descriptivo y retrospectivo que incluyó a 62 pacientes con quiste hidatídico pulmonar (QHP) sometidos a cirugía entre 2019 y 2024. La edad media fue de 39,0 ± 17,8 años y el 51,6% de los pacientes procedía de regiones del interior del país. El diámetro medio de los quistes fue de 9,3 ± 3,4 cm; el 66,1% presentó localización perihiliar y el 40,3%, quistes múltiples. La lobectomía fue el procedimiento quirúrgico más frecuente (59,7%). Las complicaciones posoperatorias se presentaron en el 12,9% de los pacientes y no se registró mortalidad perioperatoria. En conclusión, la hidatidosis pulmonar se caracterizó por una proporción importante de quistes perihiliares, múltiples y de gran tamaño. La lobectomía fue el procedimiento quirúrgico más empleado, con una baja frecuencia de complicaciones posoperatorias.

## INTRODUCCIÓN

La hidatidosis o equinococosis quística es una zoonosis parasitaria causada por la forma larvaria *de Echinococcus granulosus* y constituye un problema de salud pública, especialmente en regiones ganaderas ^1^. El ser humano actúa como hospedero intermediario accidental tras ingerir huevos del parásito, cuyas oncosferas atraviesan la mucosa intestinal y se diseminan por vía hematógena o linfática. Los quistes se localizan principalmente en el hígado (70-85%) y, en segundo lugar, en los pulmones (15-25%) ^1^^,^^2^. El quiste hidatídico pulmonar (QHP) puede alcanzar gran tamaño y, aunque algunos pacientes permanecen asintomáticos, su crecimiento puede producir tos, dolor torácico, disnea y hemoptisis ^1^^-^^4^.

En la actualidad, la persistencia de esta enfermedad refleja limitaciones en los programas de control sanitario y mantiene un ciclo de transmisión endémica que afecta a más de un millón de personas, principalmente en Eurasia, Medio Oriente y América del Sur ^4^^,^^5^. Según la Organización Panamericana de la Salud (OPS), durante 2022-2023 se confirmaron 11213 casos humanos en la región, de los cuales Perú concentró el 76% (8535 casos) ^5^. Estos resultados colocan al territorio peruano como el principal foco hiperendémico del continente, donde los factores socioculturales, la crianza informal de ganado y el acceso limitado a servicios básicos de la salud rural favorecen la persistencia de la enfermedad.

El tratamiento del QHP continúa siendo un desafío. Los benzimidazoles, como el albendazol, presentan eficacia limitada y tasas de curación inferiores al 30% ^2^^,^^6^. Por ello, la cirugía constituye el tratamiento de elección para quistes grandes, sintomáticos o complicados ^1^^-^^6^. A diferencia de la localización hepática, la cirugía para QHP no tiene una estrategia quirúrgica estandarizada ^2^^,^^5^^,^^6^, debido a las propiedades mecánicas del parénquima pulmonar, que permiten un crecimiento rápido y silencioso, y a la alta susceptibilidad del quiste a complicarse por ruptura ^5^^,^^6^. Esto genera una marcada heterogeneidad técnica, desde procedimientos conservadores como la quistectomía más capitonaje hasta resecciones pulmonares mayores en casos de destrucción tisular, localización hiliar o ruptura ^(^^6^^-^^11^.

La cirugía del QHP puede asociarse con complicaciones posoperatorias (CPO), entre ellas fuga aérea, fístula broncopleural, empiema, neumotórax y reacciones anafilácticas. En Perú, las CPO fueron reportadas en 28,2% y 22,1% de los pacientes en estudios publicados en 2006 y 2008, respectivamente ^11^, siendo las más frecuentes atelectasia, hemorragia posoperatoria y fuga aérea prolongada. A nivel internacional, los quistes gigantes, la ruptura y las resecciones pulmonares se han asociado con mayor riesgo de CPO ^12^^,^^13^, mientras que infección de herida operatoria, empiema y fuga aérea prolongada se han relacionado con mayor estancia hospitalaria ^14^. En tal sentido, la evidencia nacional disponible es limitada y procede de estudios publicados hace dos décadas, lo que genera una brecha de conocimiento sobre las características clínicas, el manejo quirúrgico y los resultados perioperatorios de los pacientes tratados actualmente en el país.

Por ello, el objetivo de este estudio fue describir las características clínicas y perioperatorias de los pacientes sometidos a cirugía por hidatidosis pulmonar en el Hospital Nacional Guillermo Almenara Irigoyen (HNGAI), Lima, Perú, durante un periodo de seis años.

**MENSAJES CLAVE**

Motivación para realizar el estudio. La hidatidosis pulmonar continúa siendo un problema relevante en el Perú; sin embargo, existe limitada evidencia nacional reciente sobre las características de los pacientes sometidos a cirugía y sus resultados.

Principales hallazgos. En 62 pacientes operados entre 2019 y 2024, fueron frecuentes los quistes perihiliares, múltiples y de gran tamaño. La lobectomía fue el procedimiento quirúrgico más utilizado. Las complicaciones posoperatorias ocurrieron en el 12,9% y no se registraron muertes perioperatorias.

Implicancias en salud pública. Estos hallazgos muestran la importancia de fortalecer la detección y referencia oportuna de pacientes con hidatidosis pulmonar, para facilitar su evaluación y tratamiento especializados.

## EL ESTUDIO

Se realizó un estudio observacional, descriptivo y retrospectivo que incluyó a 62 pacientes sometidos a cirugía por quiste hidatídico pulmonar en el Servicio de Cirugía de Tórax del HNGAI, entre enero de 2019 y diciembre de 2024. Se incluyeron todos los pacientes que cumplieron los criterios de elegibilidad durante el periodo de estudio.

Se incluyeron pacientes de 14 años o más con diagnóstico de hidatidosis pulmonar e indicación de tratamiento quirúrgico. Se excluyeron pacientes gestantes, aquellos con quistes hepáticos con compromiso de la pared torácica o el mediastino y aquellos cuyas historias clínicas estaban incompletas o no contenían información sobre las principales variables del estudio.

La información se obtuvo mediante la revisión de historias clínicas utilizando una ficha de recolección de datos. Se registraron variables sociodemográficas y clínico-radiológicas, como edad, sexo, antecedentes médicos, lugar de procedencia, síntoma principal, tamaño y ruptura del quiste, pulmón y lóbulo afectados, número de quistes y localización perihiliar. Las variables perioperatorias incluyeron la técnica quirúrgica, según preservación o resección del parénquima pulmonar, reoperación, uso posoperatorio de albendazol, complicaciones posoperatorias y estancia hospitalaria.

Se definieron operacionalmente cinco variables de interés: 1) tamaño del quiste, definido como el diámetro máximo del quiste, expresado en centímetros, medido en el plano axial de la ventana mediastinal de la tomografía computarizada (TC); 2) ruptura del quiste, definida como la presencia de nivel hidroaéreo o membranas flotantes en la TC de tórax, con confirmación intraoperatoria; 3) quiste perihiliar, definido como una lesión con compromiso de estructuras vasculares o bronquiales del hilio pulmonar, identificada mediante TC y corroborada como hallazgo intraoperatorio; 4) complicaciones posoperatorias, definidas como cualquier evento adverso relacionado con la cirugía pulmonar registrado durante el periodo posoperatorio, como fístula broncopleural, sangrado posoperatorio o empiema; y 5) estancia hospitalaria total, definida como el tiempo transcurrido desde el ingreso al hospital hasta el alta médica, independientemente de la vía de ingreso (consulta externa, emergencia o referencia) o de la estancia previa en otros servicios hospitalarios, como medicina interna o neumología.

Las variables categóricas se resumieron mediante frecuencias absolutas y porcentajes. Para las variables continuas se evaluó la distribución mediante la prueba de Shapiro-Wilk y se resumieron mediante media y su desviación estándar (DE) o mediana y rango intercuartílico (RIC). El análisis estadístico se realizó con Stata BE, versión 18.0 (StataCorp, College Station, TX, EE. UU.).

El estudio fue aprobado por el Comité de Ética en Investigación del HNGAI (Carta N° 118 CIEI-OIyD-GRPA-ESSALUD-2025). La elaboración y presentación del manuscrito siguieron las recomendaciones de la guía Strengthening the Reporting of Observational Studies in Epidemiology (STROBE) ^15^.

## HALLAZGOS

Un total de 69 pacientes con diagnóstico de hidatidosis pulmonar fueron sometidos a cirugía en el Servicio de Cirugía de Tórax del HNGAI. Se excluyeron siete pacientes debido a historias clínicas incompletas o por no cumplir los criterios de elegibilidad, por lo que el análisis incluyó finalmente a 62 pacientes. La edad media fue de 39,0 ± 17,8 años y el 58,1% (36/62) no presentó antecedentes patológicos relevantes (tabla 1). La distribución según sexo fue similar, con 31 hombres (50,0%) y 31 mujeres (50,0%). Respecto al lugar de procedencia, el 51,6% (32/62) provenía de regiones del interior del país, mientras que el 48,4% (30/62) procedía de Lima.

**Tabla 1:** Características preoperatorias de los pacientes con diagnóstico de hidatidosis pulmonar sometidos a cirugía pulmonar en el Hospital Nacional Guillermo Almenara Irigoyen durante el periodo 2019-2024.

Características preoperatorias		n (%)
Edad, media (±DE)		39,0 ± 17,8
	14-24 años	15 (24,2)
	25-49	29 (46,8)
	50-64	12 (19,3)
	65 o más	6 (9,7)
Sexo		
	Femenino	31 (50,0)
	Masculino	31 (50,0)
Antecedente		
	Ninguno	36 (58,1)
	Diabetes mellitus	1 (1,6)
	Hipertensión arterial	3 (4,8)
	Cirugía por quiste hidatídico pulmonar	6 (9,7)
	Quiste hidatídico hepático	7 (11,3)
	Otros	9 (14,5)
Procedencia		
	Lima	30 (48,4)
	Provincias	32 (51,6)
Síntoma principal		
	Asintomático	3 (4,8)
	Dolor torácico	10 (16,1)
	Disnea	13 (21,0)
	Tos-vómica	23 (37,1)
	Hemoptisis	11 (17,7)
	Fiebre	2 (3,2)
Diámetro mayor (cm) del quiste, media (±DE)		9,3 ± 3,4
	≤10	37 (59,7)
	>10	25 (40,3)
Ruptura		
	No	45 (72,6)
	Sí	17 (27,4)
Pulmón afectado		
	Derecho	40 (64,5)
	Izquierdo	22 (35,5)
Número de quistes		
	Único	37 (59,7)
	Múltiple	25 (40,3)

DE: desviación estándar, cm: centímetros.

El síntoma preoperatorio más frecuente fue la tos con vómica, presente en el 37,1% (23/62) de los pacientes. Respecto a las características de los quistes, el pulmón derecho fue el más afectado (64,5%; 40/62). El 59,7% (37/62) de los pacientes presentó un quiste único y el 40,3% (25/62), quistes múltiples. Asimismo, el 59,7% (37/62) presentó quistes con un diámetro ≤10 cm, mientras que el 40,3% (25/62) presentó quistes >10 cm. Al momento del diagnóstico, el 27,4% (17/62) presentó ruptura del quiste (tabla 1).

La lobectomía fue el procedimiento quirúrgico más frecuente, realizado en el 59,7% (37/62) de los pacientes. Entre los hallazgos quirúrgicos, el lóbulo inferior derecho fue la localización más frecuente de los QHP, presente en el 33,9% (21/62) de los casos. Asimismo, se documentó localización perihiliar del quiste en el 66,1% (41/62) de los pacientes (figuras 1 y 2).

**Figura 1:** Hallazgos tomográficos y distribución anatómica del quiste hidatídico pulmonar. A) Tomografía axial (ventana pulmonar) que muestra un quiste roto con membranas colapsadas (signo del camalote). B) Tomografía axial (ventana mediastínica) con presentación múltiple. C) Reconstrucción coronal que destaca la localización perihiliar bilateral y su relación con el mediastino. D) Esquema de la distribución porcentual según el lóbulo afectado (LSD: lóbulo superior derecho; LM: lóbulo medio; LID: lóbulo inferior derecho; LSI: lóbulo superior izquierdo; LII: lóbulo inferior izquierdo).

**Figura 2:** Hallazgos intraoperatorios de piezas quirúrgicas por quiste hidatídico pulmonar. A) Abordaje intraoperatorio mediante toracotomía que muestra la apertura de la cavidad pulmonar y la aspiración del contenido de quiste hidatídico. B) Exposición de la cavidad residual colapsada tras la apertura de la capa periquística. C) Membrana hidatídica germinativa y cuticular blanquecina.

Durante el periodo posoperatorio, el 75,8% (47/62) de los pacientes recibió tratamiento complementario con albendazol. Las complicaciones posoperatorias se presentaron en el 12,9% (8/62) de los pacientes, siendo la fístula broncopleural y la infección del sitio operatorio las más frecuentes. El 14,5% (9/62) requirió reoperación debido principalmente a hemorragia, fístula broncopleural o empiema. Un paciente con fístula broncopleural requirió dos reoperaciones: una para el cierre de la fístula y otra para el manejo del empiema. La mediana de estancia hospitalaria total fue de 12 días (RIC: 6-23 días). No se registraron muertes perioperatorias relacionadas con la cirugía por quiste hidatídico pulmonar (tabla 2).

**Tabla 2:** Características posoperatorias de los pacientes con diagnóstico de hidatidosis pulmonar sometidos a cirugía pulmonar en el Hospital Nacional Guillermo Almenara Irigoyen durante el periodo 2019-2024.

Características posoperatorias		n (%)
Técnica quirúrgica		
	Lobectomía	37 (59,7)
	Quistectomía + capitonaje	22 (35,5)
	Lobectomía + quistectomía	3 (4,8)
Lóbulo pulmonar afectado		
	Lóbulo superior derecho	13 (21,0)
	Lóbulo medio	7 (11,3)
	Lóbulo inferior derecho	21 (33,9)
	Lóbulo superior izquierdo	7 (11,3)
	Lóbulo inferior izquierdo	14 (22,5)
Quiste perihiliar		
	No	21 (33,9)
	Sí	41 (66,1)
Reoperación		
	No	53 (85,5)
	Sí	9 (14,5)
Uso de albendazol posoperatorio		
	No	15 (24,2)
	Sí	47 (75,8)
Complicación		
	No	54 (87,1)
	Sí	8 (12,9)
Tipo de complicación		
	Ninguna	54 (87,1)
	Fístula broncopleural	2 (3,2)
	Empiema	1 (1,6)
	Infección de sitio operatorio	2 (3,2)
	Sangrado	1 (1,6)
	Sepsis	2 (3,3)
Días hospitalización, mediana y rango intercuartil		12 (6-23)

## DISCUSIÓN

En este estudio, la hidatidosis pulmonar afectó principalmente a adultos jóvenes, con una edad media de 39,0 ± 17,8 años y predominio de pacientes sin antecedentes patológicos relevantes. Estos hallazgos son comparables con los reportados en el Hospital Nacional Cayetano Heredia, donde la edad media fue de 32,9 ± 18,6 años ^16^, y en el Hospital Clínico Regional de Concepción, Chile, donde fue de 34,4 ± 19,0 años ^9^, aunque difieren de lo descrito en el Hospital Nacional Dos de Mayo, con una media de 21,6 ± 14,1 años ^10^^,^^11^.

No se observaron diferencias en la distribución según sexo, con igual proporción de hombres y mujeres. Este resultado es similar al de otros estudios nacionales, en los que la proporción de hombres osciló entre 50,4% y 54,0% ^10^^,^^11^, pero difiere de algunas series internacionales, donde se ha reportado un predominio masculino de hasta 62,7% ^12^^-^^14^. Respecto a la procedencia, el 51,6% de los pacientes provenía de regiones del interior del país y el 48,4% de Lima, mientras que estudios nacionales previos reportaron que entre 77,0% y 79,0% de los casos procedían de zonas rurales y presentaban antecedentes de crianza intradomiciliaria de perros ^10^^,^^11^^,^^16^. Esto sugiere que los pacientes que actualmente residen en Lima correspondan a casos de migración interna procedentes de zonas endémicas.

Es importante señalar que una proporción de los pacientes residentes en Lima Metropolitana tuvieron contacto con regiones altoandinas y endémicas del Perú, como Huánuco, Pasco, Junín, Huancavelica, Apurímac, Cusco, Arequipa y Puno. Este patrón es compatible con la influencia de la movilidad poblacional desde áreas endémicas hacia Lima; sin embargo, debido a que no se dispuso de información detallada sobre residencia previa, duración de la exposición, contacto con perros o actividades pecuarias, no es posible establecer el lugar ni el momento probable de adquisición de la infección. Estos hallazgos resaltan la importancia de fortalecer las medidas de prevención y control de la hidatidosis en áreas endémicas ^10^^,^^11^^,^^16^.

En cuanto a la presentación clínica, la tos y la vómica fueron los síntomas predominantes. Asimismo, el 27,4% de los pacientes presentó ruptura del quiste al momento del diagnóstico. La ruptura del QHP puede producir comunicación con el árbol bronquial y desencadenar una respuesta inflamatoria local, lo que favorece la aparición de manifestaciones como tos, vómica, dolor torácico y hemoptisis ^17^^,^^18^. La proporción de quistes rotos observada en nuestra serie podría estar relacionada con lesiones de mayor tamaño o con una evolución prolongada antes del tratamiento.

El diámetro medio de los quistes fue de 9,3 ± 3,4 cm, similar al reportado en una serie de Chile (9,1 ± 4,9 cm) ^9^ y superior al descrito en series de Turquía, donde osciló entre 4,0 y 6,0 cm ^12^. Aunque la mayoría de los pacientes presentó un quiste único (59,7%), en concordancia con estudios nacionales previos ^10^^,^^11^, el 40,3% presentó quistes múltiples, lo que puede estar relacionado con diferentes mecanismos de diseminación o exposición al parásito y constituye un aspecto clínicamente relevante por la mayor complejidad que puede representar para el abordaje terapéutico ^2^^,^^10^^,^^11^^,^^19^.

Desde el punto de vista quirúrgico, la lobectomía fue el procedimiento más frecuente (59,7%), en concordancia con series nacionales previas ^10^^,^^11^, pero contrasta con reportes de Medio Oriente y Sudamérica, donde predominan enfoques conservadores como la quistectomía con capitonaje (83,7%) ^8^^,^^9^^,^^20^. Esta necesidad de recurrir a resecciones mayores en nuestro medio refleja una mayor proporción de quistes complejos, perihiliares, rotos o con destrucción parenquimatosa que impide la preservación pulmonar ^10^^,^^11^^,^^16^^,^^21^, concentrándose principalmente en el lóbulo inferior derecho (33,9%) debido a factores anatómicos y de perfusión pulmonar ^2^^,^^6^^,^^22^.

Lejos de ser un desafío estrictamente técnico intraoperatorio, esta alta proporción de formas complejas constituye el reflejo directo de un problema latente de salud pública vinculado al diagnóstico tardío, el retraso en las referencias y los patrones de migración interna en el país. Frente a este escenario el sistema de salud peruano requiere una transición desde el enfoque puramente recuperativo hacia estrategias de captación oportuna que inicien desde la edad escolar y adolescencia en zonas endémicas. Asimismo, en el ámbito urbano, es importante la implementación de protocolos de tamizaje epidemiológico mediante radiografía de tórax o ecografía en atención primaria y en evaluaciones anuales de salud ocupacional, enfocados en personas con antecedente de residencia o exposición pecuaria en la juventud. Esta intervención permitiría la detección precoz del QHP, reduciendo el número de cirugías pulmonares resectivas ^5^^,^^23^, acortando la estancia hospitalaria y mitigando el impacto socioeconómico negativo que esta enfermedad genera sobre la población económicamente activa de la seguridad social.

Respecto al manejo posoperatorio, el 75,8% de los pacientes recibió albendazol como tratamiento complementario, en concordancia con el empleo de benzimidazoles en determinados escenarios de la equinococosis quística ^7^^,^^23^. El 24,2% restante no recibió albendazol debido a la presencia de QHP rotos con evacuación de las membranas antes del procedimiento quirúrgico, escenario en el que el beneficio del tratamiento antiparasitario sería limitado ^2^^,^^5^^,^^7^^,^^23^. Por otro lado, las complicaciones posoperatorias se presentaron en el 12,9% de los pacientes, proporción inferior al 15,3 a 27,1% reportado en otras series ^10^^,^^11^^,^^24^^,^^25^. Estos resultados favorables de nuestro estudio deben interpretarse considerando el carácter descriptivo y la experiencia del centro en el manejo quirúrgico del QHP.

Este estudio presenta algunas limitaciones inherentes a su diseño observacional descriptivo y retrospectivo, basado en la revisión de historias clínicas. En primer lugar, la disponibilidad y calidad de los registros clínicos y radiológicos pudieron ocasionar sesgo de información. Asimismo, no se dispuso de información detallada sobre potenciales factores de exposición relacionados con la transmisión de la enfermedad, como el tiempo de residencia en zonas rurales o endémicas, la convivencia con perros y la exposición a actividades pecuarias, lo que limitó una mejor caracterización epidemiológica de los pacientes. En segundo lugar, al tratarse de un estudio realizado en un único centro de referencia, sus resultados no serían generalizables a pacientes atendidos en otros establecimientos. Aunque se incluyó a todos los pacientes que cumplieron los criterios de elegibilidad durante el periodo de estudio (n=62), el tamaño de la muestra limitó la posibilidad de explorar algunas asociaciones. Finalmente, la ausencia de un protocolo quirúrgico estandarizado durante el periodo evaluado pudo introducir variabilidad en la selección de la técnica quirúrgica y en los resultados perioperatorios. Futuros estudios multicéntricos con diseños analíticos podrían comparar los resultados de las diferentes estrategias quirúrgicas, particularmente la lobectomía y los procedimientos conservadores, e identificar los factores asociados con la elección de la técnica quirúrgica y la aparición de complicaciones posoperatorias.

En conclusión, la hidatidosis pulmonar en nuestra serie afectó principalmente a adultos jóvenes, en su mayoría sin antecedentes patológicos relevantes, y se caracterizó por una proporción importante de quistes perihiliares, múltiples y de gran tamaño. La lobectomía fue el procedimiento quirúrgico más empleado, con una baja frecuencia de complicaciones posoperatorias y ausencia de mortalidad perioperatoria. Estos hallazgos reafirman la necesidad de optimizar mecanismos de referencia oportuna hacia centros especializados, implementar programas de tamizajes dirigidos a la población joven en áreas endémicas e incorporar evaluaciones ocupacionales periódicas en entornos urbanos receptores de migración.
